# The infralimbic mineralocorticoid blockage prevents the stress-induced impairment of aversive memory extinction in rats

**DOI:** 10.1038/s41398-022-02118-2

**Published:** 2022-08-24

**Authors:** Kairo Alan Albernaz-Mariano, Carolina Demarchi Munhoz

**Affiliations:** Department of Pharmacology, Institute of Biomedical Science, University of Sao Paulo, Sao Paulo, 05508-000 Brazil

**Keywords:** Learning and memory, Psychiatric disorders

## Abstract

Individuals deal with adversity and return to a normal lifestyle when adversity ends. Nevertheless, in specific cases, traumas may be preceded by memory distortions in stress-related malaises, and memory extinction impairment is strictly associated with the symptoms of post-traumatic stress disorder. Glucocorticoids (GCs), the central stress mediator, target mineralocorticoid (MR) and glucocorticoid (GR) receptors and coordinate stress responses. Despite MRs being present in brain regions essential to cognition, emotions, and initial stress processing, such as the medial prefrontal cortex (mPFC), most studies attempt to elucidate the stress-induced deleterious actions of GCs via GR. Therefore, it is necessary to understand the relationship between stress, infralimbic mPFC (IL), and memory and how MR-mediated intracellular signaling influences this relationship and modulates memory extinction. We observed that acutely restraint-stressed male Wistar rats showed high corticosterone (CORT) levels, and previous intra-IL-spironolactone administration (a selective MR antagonist) decreased it 60 min after the stress started. Intra-IL-CORT118335, a novel mixed MR/GR selective modulator, increased CORT throughout stress exposure. Ten days after stress, all rats increased freezing in the memory retrieval test and acquired the aversive contextual memory. During the extinction test, intra-IL injection of spironolactone, but not CORT118335, prevented the stress-impaired memory extinction, suggesting that the IL-MR activity controls CORT concentration, and it is crucial to the establishment of late extinction impairment. Also, the concomitant GR full activation overrode MR blockage. It increased CORT levels leading to the stress-induced extinction memory impairment, reinforcing that the MR/GR balance is crucial to predicting stress-induced behavioral outcomes.

## Introduction

Stressful or traumatic experiences throughout life are inevitable [1]. In these aversive situations, physiological changes occur, and the organism must return to a healthy, controlled condition when adversity ends. However, stress duration (acute and transient or chronic and prolonged) dictates the organism’s adaptation and exhaustion dynamics, influencing behavioral and memory processes [2, 3].

Glucocorticoid (GCs) hormones (cortisol in humans and corticosterone (CORT) in rodents) are the central modulators of stress responses. They bind to and activate the mineralocorticoid and glucocorticoid receptors (MR and GR, respectively) in the central nervous system, triggering nongenomic and genomic responses [4] and altering brain cell functions [5]. GCs, released by the adrenal gland cortex during stress, play an essential role in mediating behavioral strategies and adaptation and facilitating learning through aversive memory formation [6–11]. However, most of the findings suggest that GCs actions in the brain are commonly associated with harmful and disruptive effects on memory formation [12–18]. Recently, studies showed that acute stress promotes late (10 days post-stress) physiological and behavioral changes, inducing anxiety-like behavior and aversive memory extinction impairment [17, 19]. The MR and GR coordinate in the brain, appraisal processes, choice of coping style, and hormonal feedback during stressful situations, facilitating learning and memory retrieval [6, 7, 20, 21]. MR is abundantly expressed in limbic areas, such as the medial prefrontal cortex (mPFC), while GR is widely distributed throughout the brain [7, 22]. The mPFC is subdivided into the cingulate, prelimbic (PrL), and infralimbic (IL) cortex [23] and modulates learning and decision-making functions with different roles [24]. Furthermore, the mPFC plays a role in neuroendocrine modulation during stress response [25], with the PrL inhibiting the hypothalamus-pituitary-adrenal axis (HPA, negative feedback) and the IL activating it (positive feedback) [26–29].

Two-hour of restraint stress is sufficient to generate anxiety-like behavior in rats 10 days later [30, 31], and stressed animals show increased synaptic plasticity in mPFC [32–37]. Also, the mPFC has an essential role in different stages of memory [38, 39], for example, the consolidation of extinction learning or recalling contexts [40, 41], and injuries in the IL-mPFC impair memory extinction [24, 40, 42–45].

MR is critical in modulating attentional and cognitive processes associated with aversive situations [46, 47]. Although the MR blockade or ablation in the forebrain impairs behavioral adaptations and aversive extinction and reduces the expression of some unconditioned aversive states [48, 49], the late effect of limbic MR on aversive memory extinction remains largely unknown. Thus, considering the presence of MR and GR in the mPFC [29, 50–52] and the MR implication in memory formation, behavioral adaptations, and fear extinction [48, 53, 54], it would be plausible that MR influences late mnemonic processes after previous acute stressful situations. Hence, this study sought to determine whether the late (10 days post-stress) aversive memory extinction impairment was related to the GCs actions during acute restraint stress and whether the infralimbic MR would play a role.

## Experimental procedures

### Subjects

A total of 130 male Wistar rats (60 days old at the beginning of the experiments) from the Facility for SPF Rat Production at the Institute of Biomedical Sciences - ICB at the University of Sao Paulo and maintained in the Facility of Pharmacology Department – Unit I was used as experimental subjects. All the experiments were conducted under the standards of the Ethics Committee for Animal Use of the ICB (85/2016) and Animal Research: Reporting of In Vivo Experiments (ARRIVE) guidelines [55].

The animals were randomly housed (four per home cage) in standard polypropylene cages (30 x 40 x 18cm), under controlled temperature (23 ± 2 °C) and light (light/dark cycle of 12/12 h, lights on at 7:00 am), and access to food and water *ad libitum*, except during the experiments. We took all efforts to minimize animal suffering and reduce the number of animals to the minimum required to detect significant statistical effects.

### Drugs

Spironolactone (MR antagonist; Tocris Bioscience, USA) was administered intra-IL-mPFC at 10 ng.μL^−1^. The drug was dissolved in a sterile vehicle (1% ethanol diluted in PBS) and used as the control. The CORT118335 (mixed MR/GR selective modulator; MR antagonist + GR agonist, Corcept Therapeutics, Menlo Park, CA-USA) was administered intra-IL-mPFC at 10 ng.μL^−1^. The drug was dissolved in a sterile vehicle (0.5% chloroform diluted in PBS) and used as a control. The doses were chosen according to previous studies [49, 56, 57].

### Surgery and microinjection

The animals were anesthetized with ±2-3% isoflurane (Cristalia, Itapira-SP, Brazil) in 2 L/min of O_2_ in a vaporizer (Surgivet Model 100 Forane Vaporizer, USA) and underwent stereotaxic surgery (EFF331 Insight Equipamentos, Brazil) for the bilateral implantation of guide cannulas (0.65-gauge x 12 mm) into the IL-mPFC [anteroposterior = +2.5 mm; mediolateral = ±3.1 mm; dorsoventral = −4 mm, angled 35°] [58]. The cannulas were fixed to the skull surface with dental acrylic and jeweler’s screws. To prevent contamination and occlusion, a dummy cannula (0.5-gauge stainless steel wire) was placed inside each cannula. The animals received an injection of anti-inflammatory ketoprofen (5 mg.kg^−1^, s.c. for 3 consecutive days) during post-operative recovery (4 days) [59, 60].

On the restraint stress day, 5 min before the restraint, rats received bilateral microinjections of spironolactone or CORT119335 or their respective vehicle. Each animal was gently immobilized throughout the procedure. The needle (30-gauge x 13 mm), connected by a polyethylene tube (PE-10) to a 10 μL Hamilton microsyringe, was inserted into the guide cannula, and 1 μL of the solution was infused over 300 s with the aid of an infusion pump (KDS100 Legacy, KD Scientific). The needles remained in place for another 60 s to prevent solution backflow.

### Experimental design

The schematic experimental design is represented in Fig. 2A and was conducted as described in Novaes, Bueno-de-Camargo and Munhoz [17]. After acclimatization (1-week), the animals were submitted to stereotactic surgery. At the end of the post-operative period, part of the animals was pharmacologically treated and restraint-stressed in the experimental room for 2 h (ST), while the other part remained in their home cages, undisturbed (nST). After stress, the animals returned to their home cages with the same cage mates for 10 consecutive days, where they remained undisturbed, except for cage cleaning. After the 10-day interval, the animals were assigned to the contextual aversive conditioning paradigm, followed by the extinction test.

### Apparatus, general procedure, and behavioral analysis

#### Restraint stress

the restrainer consisted of an opaque ventilated PVC cylinder (6 x 20cm) with one closed end. The animals were taken to the experimental room at 08:00 am and, after bilateral microinjections, were restrained for 2 h under constant monitoring.

#### Contextual aversive conditioning and aversive memory extinction

two arenas were used, a conditioning arena (CA, paired arena) and a neutral arena (UA, unpaired arena), both placed in the experimental room with the same environmental conditions. The CA (28 x 26 x 23cm) consisted of opaque white walls, a transparent lid, and stainless-steel bars on the floor connected to an electric shock generator (Insight Equipamentos, Pesquisa e Ensino, Ribeirão Preto-SP, Brazil). The UP consisted of a circular black floor (60 cm diameter) surrounded by a black EVA wall (50 cm height). During the training session (10 days after the previous acute restraint stress), each animal was individually placed into the CA for 2 min to freely explore it before the unconditioned stimulus (US) presentation (1 footshock of 0.5 mA, 1 s). The animals returned to their home cages 30 s after the end of the US. For the extinction paradigm, 24 h after the training session, the animals returned to the CA for a 10 min session in the absence of the US (retrieval test). This procedure was repeated for 4 consecutive days in 5 trial sessions with 24 h break between them. Twenty-four hours after the last extinction session, the animals returned to the CA for 10 min for the aversive memory extinction test.

To analyze the pairing of the US to the context, part of the animals was individually placed, 24 h after training, into the UA to freely explore for 10 min, followed by 5 re-exposures with 24 h intervals between them. Twenty-four hours after the fifth re-exposure, the animals returned to the CA. This control confirmed whether changes in behavior during aversive memory extinction were related to time-lapsing or the re-exposure to the conditioning arena in the absence of the unconditioned stimulus.

Animal behavior was recorded using a webcam (Logitech C920 HD Pro, Lausanne, Switzerland) from the top of the chamber. The percentage of freezing behavior (total freezing time/600 s) × 100) was used to measure aversive memory. We considered freezing as the complete immobility of the animal (including vibrissae and sniffing movements) except for respiration-related movements [61]. It was scored automatically using the ANY-maze software (6.33 version, Stoelting, IL, USA). Freezing was analyzed during the training session (2 min before and 30 s after the unconditioned stimulus presentation, Pre-US or Post-US, respectively) and 10 min of each exposure. After each exposure, the arenas were cleaned with 5% alcohol to avoid olfactory cues. All tests were conducted between 09:00 am and 2:00 pm.

### Euthanasia and sample preparation

#### Transcardial perfusion and sample preparation

the animals were anesthetized with isoflurane and transcardially perfused using a peristaltic pump (Cole Parmer) with 200 mL of saline solution (0.9%) for 7 min, followed by 400 mL of formaldehyde solution (4%) in sodium phosphate buffer (pH 7.4) at 4 °C for 14 min. Next, their brains were removed and stored in formalin solution (4%). Twenty-four hours later, the brains were transferred to a sucrose solution (30%). After sunk, they were stored at −80 °C for future use.

#### Histological procedure

the coronal brain sections (40 μm thickness) were collected using a cryostat at −25 °C (Leica CM1850UV, Leica Biosystems, Germany). They were verified using a stereomicroscope (Tecnival-Prolab, São Paulo-SP) and compared to the rat brain atlas [58]. The visualization of the gliosis indicated the microinjection sites. The animals with microinjection sites outside the IL-mPFC (approximately 5%) were excluded from the analysis (Fig. S1A-C, supplementary information).

### CORT analysis

**Fig. 1:**
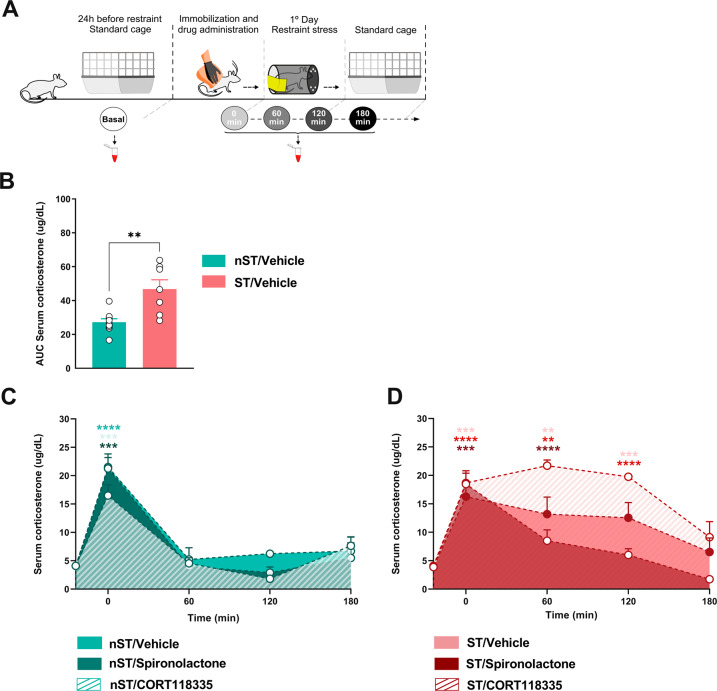
Corticosterone concentration in restraint-stressed animals after infralimbic MR and GR modulation. Schematic representation of the experimental design (**A**). The area under the curve (AUC) of serum corticosterone concentration in restraint-stressed animals (ST) (**B**). Serum corticosterone concentration curve in unstressed animals (nST) subjected to IL-mPFC MR and GR modulation (**C**). Serum corticosterone concentration curve in stressed animals (ST) subjected to IL-mPFC MR and GR modulation (**D**). Results are represented as mean ± SEM (*n* = 7–9 animals per group in B; *n* = 7–12 animals per group in C; *n* = 9-12 animals per group in **D**). T-test (**B**). Two-way mixed-effects model ANOVA followed by Tukey’s test (C and D). In B, significant differences are indicated as ** (*p* < 0.01) vs nST/Vehicle. In C and D, significant differences are indicated as **, ***, **** (*p* < 0.01, *p* < 0.001, *p* < 0.0001, respectively) vs baseline. Spironolactone and CORT118335 at dose 10 ng.μL^−1^.

Blood was sampled from the caudal vein at five different time points and mainly collected during experiments 3 and 4. A sample was collected 24 h before acute restraint stress as the baseline. On the day of restraint, 4 collections were performed within 60 min intervals between them (Fig. 1A). The first collection was performed immediately after the animals were placed into the restrainer, and the last one was performed 60 min after the end of the restraint. Blood was collected in non-heparinized tubes, allowed to clot at room temperature for 30 min, and then centrifuged at 4000 rpm for 10 min at 4 °C. The serum was collected and stored at −80 °C. According to the manufacturer’s specifications, quantifications were performed using the CORT enzyme immunoassay Kit (ELISA, Enzo Life Sciences).

### Statistical analysis

For CORT analysis, the differences between the experimental and the control groups were detected by unpaired T-test. Two-way mixed-effects model ANOVA was used when more than 2 experimental groups were analyzed followed by *post-hoc* Dunnett’s or Tukey’s tests. In these situations, group (stress + treatment) and time were between-subject factors. For behavioral analysis, data were treated as repeated measures, and the differences between the experimental and the control groups were detected by unpaired T-test. Two-way mixed-effects model ANOVA or two-way ANOVA was used when more than 2 experimental groups were analyzed followed by *post-hoc* Tukey’s test. In these situations, condition (stress), group (stress + treatment) and time were between-subject factors.

Data analysis and quantification were blind for all experiments. GraphPad Prism 9 software was used for the statistical analyses, and data are presented as mean ± SEM. The minimum level of statistical significance was set up at *p* < 0.05. Sample size and animal numbers were estimated based on previous studies. Outliers were excluded from the analysis using the ROUT method. Table S1 in the supplementary information indicates the exact sample size per group in each experiment.

## Results

### Infralimbic MR antagonism prevents the elevation of acute restraint stress-induced CORT concentration

Considering that MR and GR modulation alters stress responses, we sought to determine their effects on CORT release. The experimental design is depicted in Fig. 1A. Our results confirmed that 2 h of restraint stress increased CORT concentration time-dependently, peaking immediately at the beginning of stress (0 min) and returning to basal 180 min after stress ended (Fig. 1D).

During the baseline period (24 h before stress), one-way ANOVA showed no significant effect on CORT concentration (condition [F (_1, 63_) = 3.80; *p* > 0.05], treatment [F (_3, 63_) = 0.52; *p* > 0.05] and interaction between factors [F (_3, 63_) = 1.07; *p* > 0.05]) (Fig. S2A, supplementary data), indicating that all animals had similar basal CORT levels. Thus, we used a single baseline, calculated from the sum of the groups’ mean, to analyze the next results. A one-way ANOVA revealed significant effect of the initial collection period (0 min) on CORT (time [*F*_(8, 71)_ = 5.103; *p* < 0.0001]). All groups showed an increase in CORT compared to baseline (confirmed by Dunnett’s test). An area under the curve (AUC) analysis confirmed that 2 h of restraint stress increased CORT serum levels compared to nST animals (T-test [t _(18)_ = 3.226; *p* < 0.01]) (Fig. 1B).

When analyzing CORT release in the nST animals, two-way mixed-effects model ANOVA revealed a significant effect of the collection period (time [*F*_(1.981, 25.76)_ = 49.32; *p* < 0.0001], but absence for the treatment [*F*_(1.439, 18.70)_ = 3.378; *p* > 0.05] or interaction between factors [*F*_(2.399, 0.799)_ = 1.027; *p* > 0.05]), indicating that IL-mPFC drug infusion did not modulate CORT release in those animals (Fig. 1C). However, all nST animals showed higher CORT serum levels at the first collection (0 min) than the baseline (confirmed by Tukey’s test), indicating a possible effect of animal experimental manipulations (Fig. S2B, supplementary data).

For the ST group, two-way mixed-effects model ANOVA indicated a significant effect of treatment over time (treatment [F (_1.808, 69.29_) = 12.25; *p* < 0.0001], time [F (_2.592, 74.52_) = 39.34; *p* < 0.0001] and interaction between factors (F (_2.727, 26.14_) = 3.089; *p* < 0.05). In the ST/Vehicle group, as mentioned, CORT serum peaked immediately after restraint, maintaining elevated through the 2 h restraint stress. CORT serum levels returned to baseline 1 h after stress ended (confirmed by Tukey’s test). Intra-IL-mPFC spironolactone MR blockage did not interfere with the CORT serum peak; however, these hormone levels decreased 1 h after the restraint initiation compared to ST/Vehicle group, returning to the basal levels while still during restraint (confirmed by Tukey’s test). Intra IL-mPFC MR and GR simultaneous modulation by CORT118335, on the other hand, further increased CORT release during restraint stress compared to ST/Vehicle group, which was sustained during restraint, returning to baseline 1 h after stress (confirmed by Tukey’s test) (Fig. 1D). Our results show that IL-mPFC MR/GR activation balance is crucial to modulating the restraint-stress-induced HPA axis activation and CORT release.

### Acute restraint stress impairs the late memory extinction process (measured 10 days post-stress)

**Fig. 2:**
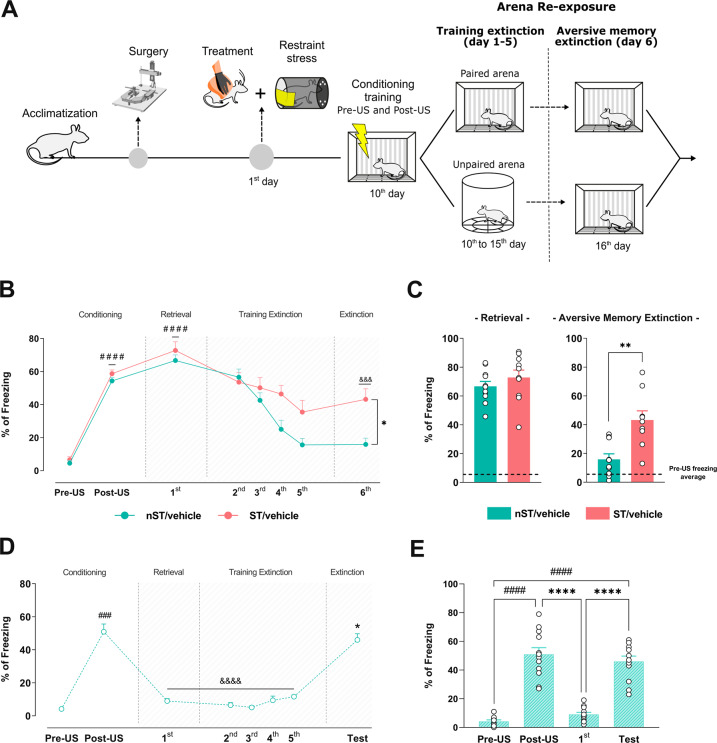
Previous acute restraint stress impairs the late memory extinction process. Schematic representation of the experimental design (**A**). Percentage of freezing over days in the extinction paradigm in previously 2 h restraint-stressed rats (**B**). Column graph representing the percentage of freezing response of stressed (ST) and unstressed (nST) animals during retrieval and aversive memory extinction tests in the absence of extinction training (**C**). Percentage of freezing over days in the extinction paradigm in nST rats submitted to an unpaired context (**D**). Column graph representing the percentage of freezing response during Pre-US, Post-US, retrieval, and aversive memory extinction tests of rats submitted to an unpaired context (**E**). Results are represented as mean ± SEM (n = 10-11 animals per group in B and C; *n* = 12 animals per group in **C** and **D**). Two-way mixed-effects model ANOVA (B and D) followed by Tukey’s test, T-test (**C**), and one-way ANOVA (D and E) followed by Tukey’s test. In B, #### (*p* < 0.0001vs their respective group in Pre-US period, &&& (*p* < 0.001) vs to their respective group during retrieval test, and * (*p* < 0.05) vs nST/Vehicle group to aversive memory extinction test. In C, ** (*p* < 0.01) vs nST/Vehicle during the aversive memory extinction test. In D, ### (*p* < 0.001) vs Pre-US period, &&&& (*p* < 0.0001) vs aversive memory extinction test, and * (*p* < 0.05) vs retrieval test. In E, #### (*p* < 0.0001) vs Pre-US period, **** (*p* < 0.0001 or *p* < 0.0001) vs retrieval test.

To investigate the stress-induced late effect on the aversive memory extinction, rats were stressed and posteriorly (10 days later) submitted to the contextual aversive conditioning and the extinction paradigm in the paired or unpaired arena, as illustrated in Fig. 2A. Two-way mixed-effects model ANOVA revealed a significant effect on the percentage of freezing in ST animals over days [condition (*F*_(1, 10)_ = 10.80; *p* < 0.05), time (*F*_(7, 70)_ = 44.50; *p* < 0.0001) and interaction between factors (*F*_(7, 59)_ = 3.23; *p* < 0.001)]. ST and nST animals showed an increase in the percentage of freezing after contextual aversive conditioning (post-US period) and after 24 h in the retrieval test (day 1) (confirmed by Tukey’s test), indicating that both groups expressed defensive behavior and formed long-term memory (Fig. 2B, C).

During the aversive memory extinction test, the previous stress further increased the freezing percentage compared to nST animals. Still, both groups showed a lower percentage of freezing compared to day 1, indicating a late stress-induced aversive memory extinction impairment (Fig. 2B), better observed when comparing the freezing percentage on the first (retrieval memory) and sixth day (aversive extinction memory test) between the experimental groups (T-test on days 1 [t _(19)_ = 1.00; *p* > 0.05]) and 6 [t _(17)_ = 3.75; *p* < 0.01]) (Fig. 2C). Our results indicated that previous stress specifically impaired the extinction of the aversive memory (measured on day 6) without altering the acquisition and consolidation of aversive memory.

Regarding the unpaired group, one-way ANOVA showed a significant effect of time on the percentage of freezing [time (*F*_(7, 88)_ = 57.73; *p* < 0.0001)]. The unpaired group presented an increase of freezing during the post-US period in the paired arena when compared to pre-US but did not during days 1 to 5 in the unpaired arena, indicating that the expressed defensive behavior was context-dependent and did not generalize to other contexts (confirmed by Tukey’s test) (Fig. 2D). In addition, when submitted to the paired arena during the aversive memory extinction test, the unpaired group increased freezing compared to the retrieval test (confirmed by Tukey’s test). This data suggests that the decrease in freezing was fundamentally related to the successive re-exposures to the paired arena and not because of the time-lapse between sessions (Fig. 2D). The same observations were confirmed in the absence of a training period (comparing pre-US, post-US, days 1 and 6) was performed between the experimental groups [one-way ANOVA; time (*F*_(3, 44)_ = 60.24; *p* < 0.0001)] (confirmed by Tukey’s test) (Fig. 2E).

### Intra-IL infusion of the spironolactone before previous acute restraint stress prevents the late memory extinction impairment

**Fig. 3:**
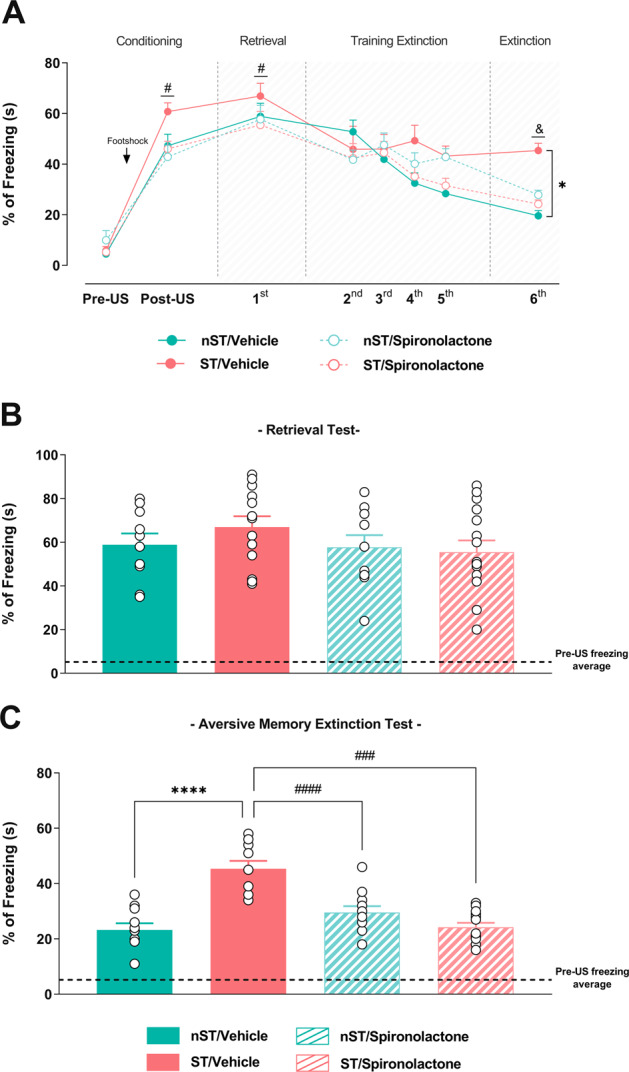
Infralimbic MR antagonism immediately before previous acute restraint stress rescues the late stress-induced memory extinction impairment. Percentage of freezing over days in the extinction paradigm in previously restraint-stressed rats subjected or not to the IL-mPFC MR antagonist with spironolactone (**A**). The column graph represents the percentage of freezing during the retrieval test in the restraint-stressed (ST) and unstressed (nST) animals treated with spironolactone (**B**). The column graph represents the percentage of freezing during the aversive memory extinction test in the restraint-stressed (ST) and unstressed (nST) animals treated with spironolactone (**C**). Results are represented as mean ± SEM (*n* = 10–15 animals per group in **B** and **C**). Two-way mixed-effects model ANOVA (B) and two-way ANOVA followed by Tukey’s tests (B). In A, # (*p* < 0.05) vs their respective group in Pre-US period, & (*p* < 0.05) vs their respective group in retrieval test, and * (*p* < 0.05) vs nST/Vehicle group in aversive memory extinction test. In **C**, **** (*p* < 0.0001) vs nST/Vehicle group, ### and #### (*p* < 0.001 and *p* < 0.0001, respectively) vs ST/Vehicle group. Spironolactone at dose 10 ng.μL^−1^.

We administrated intra IL-mPFC spironolactone 5 min before restraint stress to analyze the MR contribution in the late stress-induced aversive memory extinction impairment. Two-way mixed-effects model ANOVA indicated significant changes in the percentage of freezing over days [group (*F*_(2.40, 33.73)_ = 7.64; *p* < 0.001), time (*F*_(2.75, 38.54)_ = 61.25; *p* < 0.0001), but no interaction between factors (*F*_(5.80, 49.71)_ = 1.64; *p* > 0.05)]. Intra IL-mPFC spironolactone-treated ST and nST animals increased freezing after the conditioning period (post-US), which was maintained elevated during the retrieval test (day 1) compared to their respective group in the pre-US period (confirmed by Tukey’s test) (Fig. 3A). Therefore, all animals similarly expressed a natural defensive behavior during the aversive situation and formed long-term memory after conditioning. We also corroborated the stress-induced aversive memory extinction impairment, i.e., the maintenance of high freezing percentage during the aversive memory extinction test (day 6) (confirmed by Tukey’s test).

Spironolactone-induced infralimbic MR antagonism in ST animals prevented the impaired aversive memory extinction (confirmed by Tukey’s test) without altering the extinction capacity in the nST animals (Fig. 3A). Thus, infralimbic MR activation contributes to the late stress-induced aversive memory extinction impairment.

Comparing freezing behavior among groups and treatments specifically in the retrieval test or the extinction test, a two-way ANOVA did not indicate any significant effect in the ST animals treated with spironolactone during the first day [condition (*F*_(1, 45)_ = 0.28; *p* > 0.05), treatment (*F*_(1, 45)_ = 1.37; *p* > 0.05), or interaction between factors (*F*_(1, 45)_ = 0.91; *p* > 0.05)], but revealed significant effect during the sixth day (aversive extinction memory test) [condition (*F*_(1, 41)_ = 13.73; *p* < 0.001), treatment (*F*_(1, 41)_ = 10.78; *p* < 0.01), and interaction between factors (*F*_(1, 41)_ = 0.91; *p* < 0.0001)] (confirmed by Tukey’s tests) (Fig. 3B), evidencing that infralimbic MR activation is crucial for the stress-induced late aversive memory extinction deficit primarily.

### Intra-IL infusion of the CORT118335 before previous acute restraint stress did not modulate the late memory extinction impairment

**Fig. 4:**
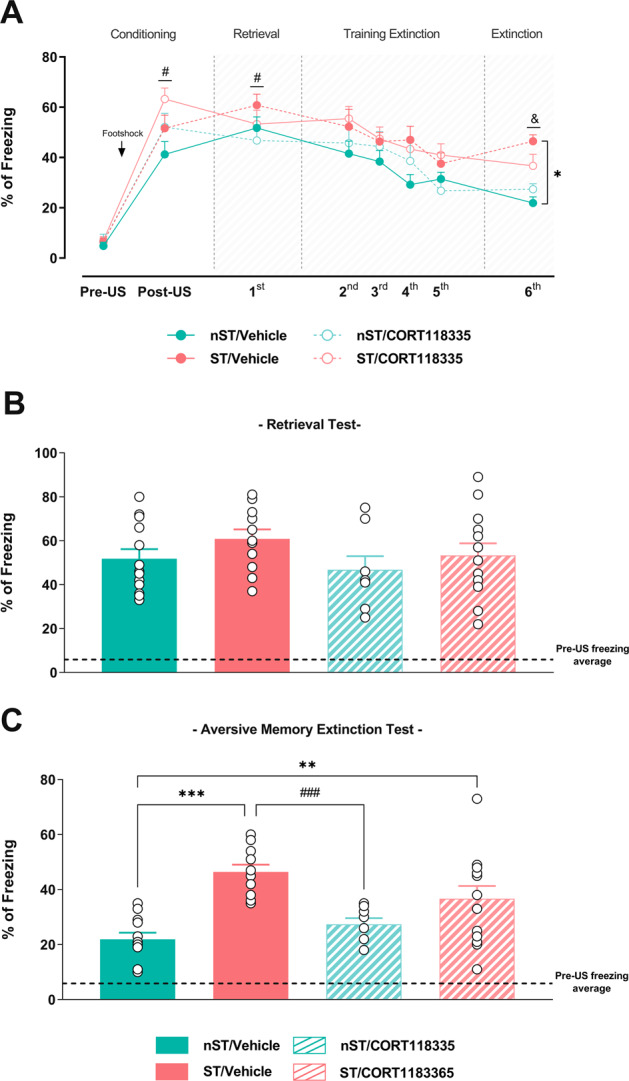
Simultaneous modulation of infralimbic MR and GR before previous acute restraint stress did not modify the late stress-induced memory extinction impairment. Percentage of freezing over days in the extinction paradigm in previously restraint-stressed rats subjected or not to the IL-mPFC mixed MR/GR selective modulator CORT118335 (**A**). Percentage of freezing during the retrieval test in the restraint-stressed (ST) and unstressed (nST) animals treated with CORT118335 (**B**). Percentage of freezing during the aversive memory extinction test in the restraint-stressed (ST) and unstressed (nST) animals treated with CORT118335 (**C**). Results are represented as mean ± SEM (n = 8-13 animals per group in B and C). Two-way mixed-effects model ANOVA (**B**) and two-way ANOVA followed by Tukey’s tests (B). In A, # (*p* < 0.05) vs their respective group in Pre-US period, & (*p* < 0.05) nST/Vehicle vs their respective group in retrieval test, and * (*p* < 0.05) vs nST/Vehicle group in aversive memory extinction test. In C, ** and *** (*p* < 0.01 and *p* < 0.001, respectively) vs nST/Vehicle group, ### (*p* < 0.001) vs ST/Vehicle group. CORT118335 at dose 10 ng.μL^−1^.

Studies have discussed the importance of MR/GR balance in stress-induced memory modulation. To analyze the MR importance under the influence of the infralimbic GR activation on late stress-induced memory effects, we administrated intra IL-mPFC CORT118335 5 min before restraint stress. Two-way mixed-effects model ANOVA revealed significant effect in the percentage of freezing over days in animals treated with CORT118335 [group (*F*_(2.18, 26.16)_ = 4.30; *p* < 0.05), time (*F*_(3.27, 39,29)_ = 58.49; *p* < 0.0001), but no interaction between factors (*F*_(5.89, 54.21)_ = 1.39; *p* > 0.05)] (Fig. 4A). Like the infralimbic spironolactone administration, CORT118335 increased freezing in ST and nST animals after the conditioning period (post-US), persisting on the retrieval test (day 1) (confirmed by Tukey’s test). This result indicated an expressed natural defensive behavior during the aversive situation and a formed long-term memory after conditioning. However, opposite to the infralimbic spironolactone treatment, CORT118335 did not prevent the stress-induced late aversive memory extinction impairment observed in the aversive memory extinction test (day 6) (confirmed by Tukey’s test).

Comparing freezing behavior among groups and treatments specifically in the retrieval test or the extinction test day, a two-way ANOVA did not revealed significant effect in the ST animals treated with CORT118335 for the first day (retrieval memory) [condition (*F*_(1, 40)_ = 1.40; *p* > 0.05), treatment (*F*_(1, 40)_ = 0.80; *p* > 0.05), or interaction between factors (*F*_(1, 40)_ = 0.28; *p* > 0.05)] (Fig. 4B), but revealed significant effect during the sixth day (aversive extinction memory test) [condition (F _(1, 38)_ = 42.04; *p* < 0.001), absence for the treatment (F _(1, 38)_ = 0.68; *p* > 0.05), but interaction between factors (*F*_(1, 38)_ = 6.42; p < 0.05)], confirming previous observations by Tukey’s tests (Fig. 4C). Our results evidence that infralimbic MR blockade alone is insufficient to modulate the stress-induced memory extinction impairment when GR is heavily occupied.

## Discussion

**Fig. 5:**
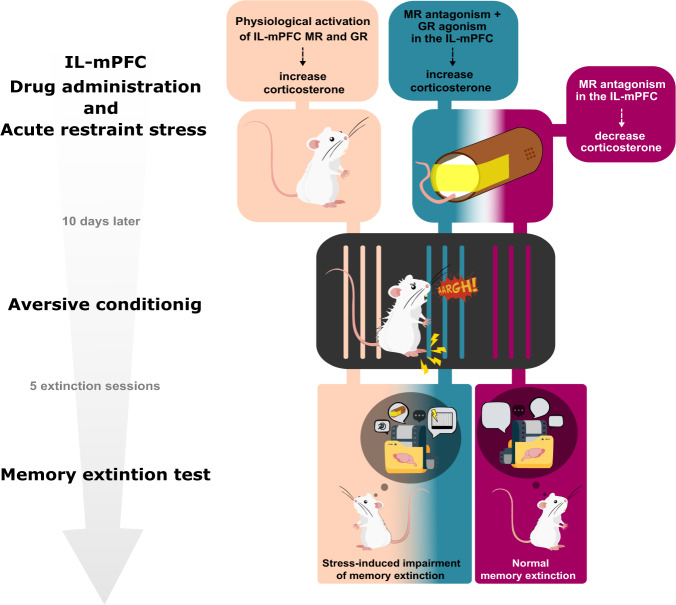
The relevance of infralimbic MR and GR activation during acute restraint stress in the stress-induced corticosterone release and late memory processes. The infralimbic MR blockage by spironolactone during restraint stress, but not the concomitant modulation of the MR by CORT118335, a mixed MR/GR selective modulator (MR antagonist + GR agonist), reduced the stress-induced corticosterone release and rescued the impaired memory extinction in rats.

The main finding of this study was to demonstrate that the infralimbic MR blockage during previous acute restraint stress, but not the concomitant modulation of the MR and GR, reduced the immediate stress-induced CORT release and rescued animals from developing the late (10 days after stress) stress-induced aversive memory extinction deficit. Our results also implicate, for the first time, the infralimbic MR activation as crucial as GRs in the late stress-induced memory extinction impairment (Fig. 5).

We found that 2 h of restraint stress in rats increased CORT concentrations, and 17 days later, after aversive conditioning, induced aversive memory extinction impairment, corroborating previous findings [17, 31, 62–64]. Other authors have shown that acute restraint stress caused late behavioral and morphological neuronal changes. For example, Mitra and Sapolsky [30] and Rao, Anilkumar, McEwen and Chattarji [65] observed, 10 days later, that 2 h of restraint stress or exogenous administration of CORT generated a late anxiety-like behavior and increased the spine density in the basolateral amygdala.

The mnemonic effects of stress-induced GCs release are still a matter of debate. C57BL/6 J mice restrained for 2 h and 6 days later received systemic administration of dexamethasone (selective GR agonist, HPA axis suppressor) after aversive conditioning showed greater aversive extinction, retention of extinction learning, and reduced *Fkbp5* mRNA expression in the amygdala [66]. Studies also indicated that higher cortisol concentrations are associated with a more significant fear acquisition in humans. However, this effect may be limited to males [67, 68], and extreme cortisol concentrations during an intense stressor may favor the amnesia of trauma-related information that affects some patients with PTSD [69]. The extinction impairment of an aversive memory is similar to the mnemonical component associated with PTSD, in which patients with trauma-related disorders have a memory extinction deficit [70, 71]. These data suggest that using the aversive memory extinction test after aversive conditioning provides essential answers to stress-related disorders. Also, the anxiety-like behaviors and memory processes such as extinction are influenced by previous events that could alter the HPA axis activity, modulating GCs sensitivity.

Fear learning and maintenance involve processes influenced by the HPA axis activity and GCs release. The effects of GCs signaling on memory formation and retrieval are complex and subject to the timing of this signaling concerning the aversive experience, and brain structure recruited [7, 21, 72]. In our study, a sustained increase in CORT for 2 h during restraint stress was necessary to generate the late extinction memory impairment since the CORT decrease 1 h after the onset of stress caused by the infralimbic MR antagonism prevented this late stress-induced effect. For the first time, these findings indicate that MR is associated with late changes in behavior, specifically in the IL-mPFC, and mediates the aversive memory extinction in previously stressed animals.

Other authors demonstrated that low systemic doses of spironolactone and dexamethasone before aversive conditioning reduced freezing time on each session, indicating an increase in the aversive memory extinction. In contrast, fludrocortisone (selective MR agonist) impaired memory extinction, high-dose of dexamethasone and mifepristone (GR antagonist in high dose) did not affect memory extinction, and spironolactone after 5 min of conditioning impaired the aversive memory extinction [73]. Also, low-dose hydrocortisone (non-selective GCs) had acute fear-reducing effects in adults with and without PTSD [74], potentially by diminishing hippocampal and amygdala activity or reducing CRH concentrations [75].

Studies suggest that the behavioral inflexibility or the constant alertness maintained in a potential threatening context can be mediated via forebrain MR and would be the critical point of animal perseverance for a particular behavior over another [76, 77]. Moreover, the balance in MR and GR activation is essential for adequately processing information related to aversive stimuli [78]. We believe that they occur due to the inhibition of MR actions or by an indirect action caused by blocking MR and the consequent displacement of GCs to activate GR, in both cases, modulating the HPA axis via mPFC. Systemic studies have shown that MR blockade by potassium canrenoate (MR antagonist) impaired the CORT negative feedback, increasing GCs release [79]. This effect is also observed with systemic or intracerebroventricular administration of spironolactone or RU 28318 (selective MR antagonist) in rats after 1 h acute restraint stress or novel-environment stress test [80–82].

Several studies have considered the importance of the balance between MR and GR activation [6, 7, 21, 83, 84]. In this sense, CORT118335 has a higher affinity for GR, and a lower affinity for MR, for which it acts as an antagonist [85]. Nguyen, Streicher, Berman, Caldwell, Ghisays, Estrada et al. [86] and Nguyen, Caldwell, Streicher, Ghisays, Balmer, Estrada et al. [87] observed that systemic administration of CORT118335 in male and female rats decreased CORT release in response to 30 min of restraint stress. Systemic injection of CORT118335 (80 mg.kg^−1^) one hour before the avoidance memory task, a test used to assess memory strength, which is potentiated by GR, increased CORT in the memory consolidation phase, similar to the classic GR antagonist RU486 [88–90]. However, studies involving the central use of simultaneous MR and GR modulators remain scarce.

Our data indicated that simultaneous modulation of infralimbic MR and GR by CORT118335 immediately elevates CORT in acutely stressed animals and is crucial for its maintenance throughout the whole stress period. In an unbalanced situation between the activation of both receptors, the increased activation of GR in the absence of MR activation may interrupt the standard CORT release, suggesting that in stressful situations, the infralimbic MR modulates the release of CORT. We hypothesized that the memory impairment would be aggravated in this situation regarding aversive memory extinction. However, we found that it remained unaltered. Therefore, it is plausible that other mechanisms and brain structures might be controlling the effects of high CORT release in the brain, such as glutamatergic, GABAergic, serotonergic, and endocannabinoid activity in neurons in the amygdala, hippocampus, and other regions of the mPFC [17, 19, 66, 91–97]. Furthermore, the binding of CORT118335 on MR showed an identical pattern of antagonism to that of eplerenone, a selective MR antagonist. However, although they are potent MR antagonists, it has been observed that spironolactone and eplerenone (and CORT118335) induced (at high concentrations) interactions between MR and co-regulatory motifs that are generated by full agonism caused by cortisol [98, 99]. This supports the notion that these antagonists can act as (partial) agonists at high concentrations or particular cellular configurations [100, 101], potentially interfering with the GCs-induced agonist effect [85]. Thus, CORT118335 promotes GR agonism and MR antagonism, consequently causing an increased GR genomic effect.

Additionally, it indirectly causes anticipation of GR effects by increasing the availability of endogenous CORT in the presence of blocked MR. In our study, the differential effects of CORT118335 concerning spironolactone could likely be due to differential effects on gene expression, which, in turn, would depend on the recruitment of distinct coregulators [102]. The imbalance in the dynamic activation of MR/GR would promote temporal disruption between MR and GR natural activation and their role in late mnemonic processes.

During stressful situations, the actions of mPFC over the HPA axis are unclear, mainly because in the same stressful situation, the mPFC subregions can overlap and respond differently, including antagonistically. Earlier studies have shown that lesions in the cingulate gyrus or PrL increased CORT in response to 20 min restraint stress, and CORT implants in these regions reversed it without affecting the HPA axis basal activity [26, 28, 103]. These findings indicate that the mPFC recruitment after a stressful stimulus sets the tone of the stress response. Moreover, IL lesions caused by ibotenic acid (glutamatergic agonist) suppress the stress-induced increase in CORT levels [104]. In line with these results, McKlveen, Myers, Flak, Bundzikova, Solomon, Seroogy et al. [29], using a virally mediated GR knockdown in the IL, observed, in rats, that IL seems to be involved in the initial emotional responses control to stressors and regulates the HPA axis during chronic stress. In general, dorsal regions of the mPFC inhibit the HPA axis function, and ventral areas, such as the IL, facilitate stress-induced HPA axis activation [103, 104]. Lau, Whiteman and Blundell [105] suggest an ideal level of GCs activation in the hippocampus. When this level exceeds or is lower than the acceptable, the consolidation of context-aversive memory can be impaired. We believe that as the hippocampus, the same occurs in IL-mPFC. Our findings align with studies showing a dose-dependent correlation between stress hormone levels and long-term potentiation (LTP). These results supported the hypothesis that corticosteroids exert a concentration-dependent biphasic influence on LTP. Other studies have corroborated the existence of an inverted-U curve function between circulating GCs levels and memory performance [54, 106, 107].

Our findings, therefore, show the relevance of infralimbic MR and GR activation during stress in the late memory extinction process and immediate CORT release. The relationship between previous acute stress and the memory extinction deficit is supported by MR and GR coordinated activity during stress, in which the GCs action in the mPFC contributes to systemic CORT elevation. As a late consequence, the extinction memory impairment is established. The infralimbic MR activity controls CORT concentration since the MR blockage during the previous restraint stress is sufficient to decrease CORT and revert the late extinction impairment. Furthermore, we also showed that the concomitant GR full activation overrides the MR blockage effect and increases CORT levels leading to the late stress-induced extinction memory impairment.

## Supplementary information

Supplemental Material.
