# Supplementary material for: The infralimbic mineralocorticoid blockage prevents the stress-induced impairment of aversive memory extinction in rats
Source: Transl Psychiatry. 2022 Aug 24;12:343. doi: 10.1038/s41398-022-02118-2 (PMC9399104; doi:10.1038/s41398-022-02118-2)
Supplement: Supplementary file 1 — Supplemental Material. [file 41398_2022_2118_MOESM1_ESM.pdf]

Table S1. **Pharmacological treatments and sample sizes of each experimental group.**

Experiment	Condition and Treatment		Nº per group
	**Experiment 1**		
Corticosterone concentration curve	nST	Non treated	n = 8
	ST	Non treated	n = 7
	nST	Vehicle	n = 12
	ST	Vehicle	n = 7
	nST	Spironolactone 10 ng.μL^{-1}	n = 14
	ST	Spironolactone 10 ng.μL^{-1}	n = 12
	nST	CORT118335 10 ng.μL^{-1}	n = 9
	ST	CORT118335 10 ng.μL^{-1}	n = 7
	**Experiment 2**		
	nST	Vehicle	n = 11
	ST	Vehicle	n = 10
	nST/Unpaired	Vehicle	n = 12
	**Experiment 3**		
Stress, behavior and memory	nST	Vehicle	n = 11
	ST	Vehicle	n = 13
	nST	Spironolactone 10 ng.μL^{-1}	n = 11
	ST	Spironolactone 10 ng.μL^{-1}	n = 14
	**Experiment 4**		
	nST	Vehicle	n = 8
	ST	Vehicle	n = 9
	nST	CORT118335 10 ng.μL^{-1}	n = 8
	ST	CORT118335 10 ng.μL^{-1}	n = 13

The numbers represent the sample size used per group. nST = unstressed and ST = stressed.

Figure S1 – **Schematic representation of the injection sites in the IL-mPFC.**

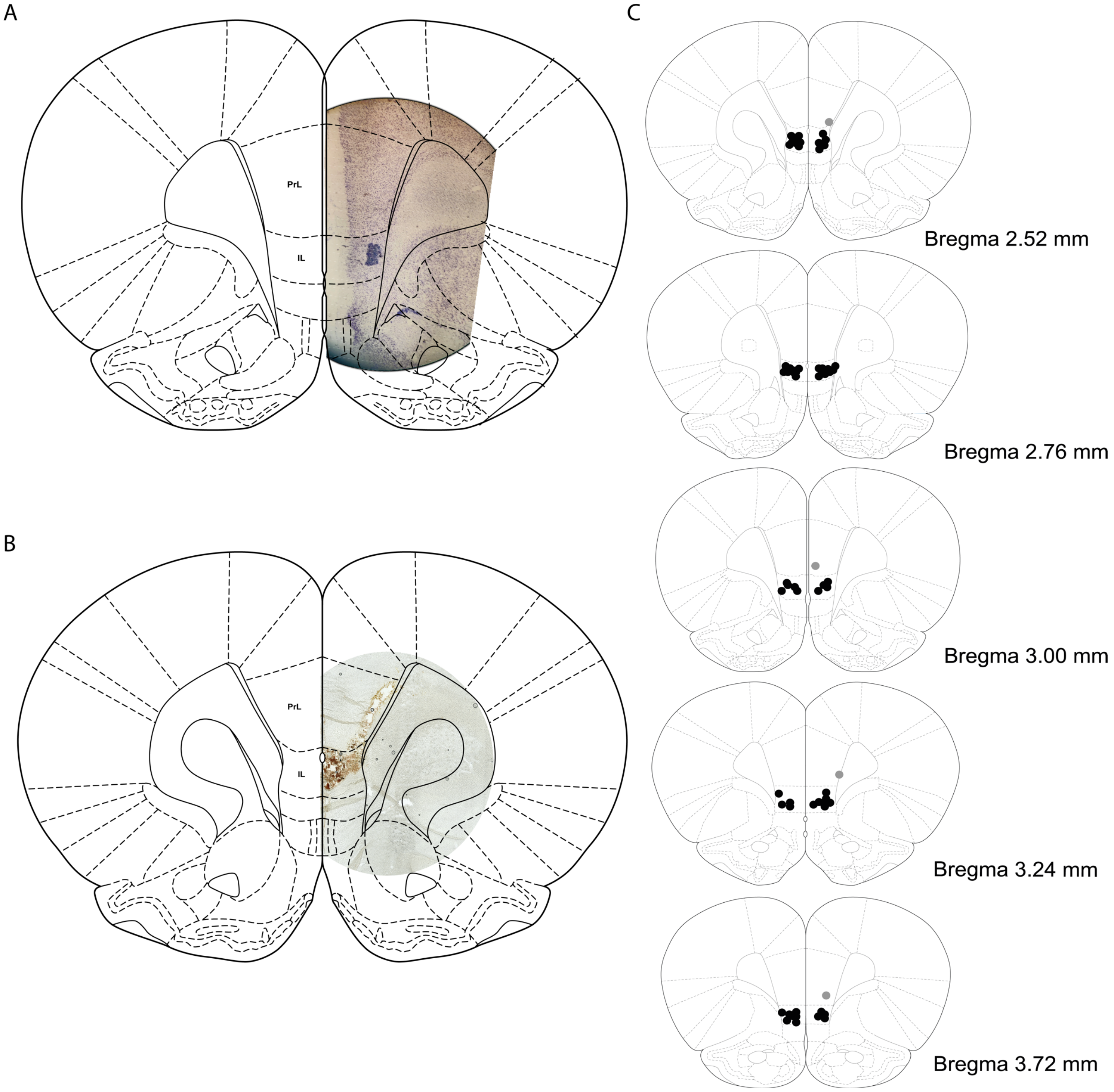

The representative subject of the coronal photomicrograph section (objective 5x) showing an injection site (**A**). The representative subject of the coronal photomicrograph section (objective 5x) showing dispersion 2 h after administration of 1 μL of vehicle solution containing 0.12% thionine (**B**). The microinfusion sites within (full circles) or outside (gray circles) of the IL. Due to the overlap, the number of points represented is less than the actual number of animals (**C**).

Figure S2 – **Corticosterone concentration in rats during the baseline period collection.**

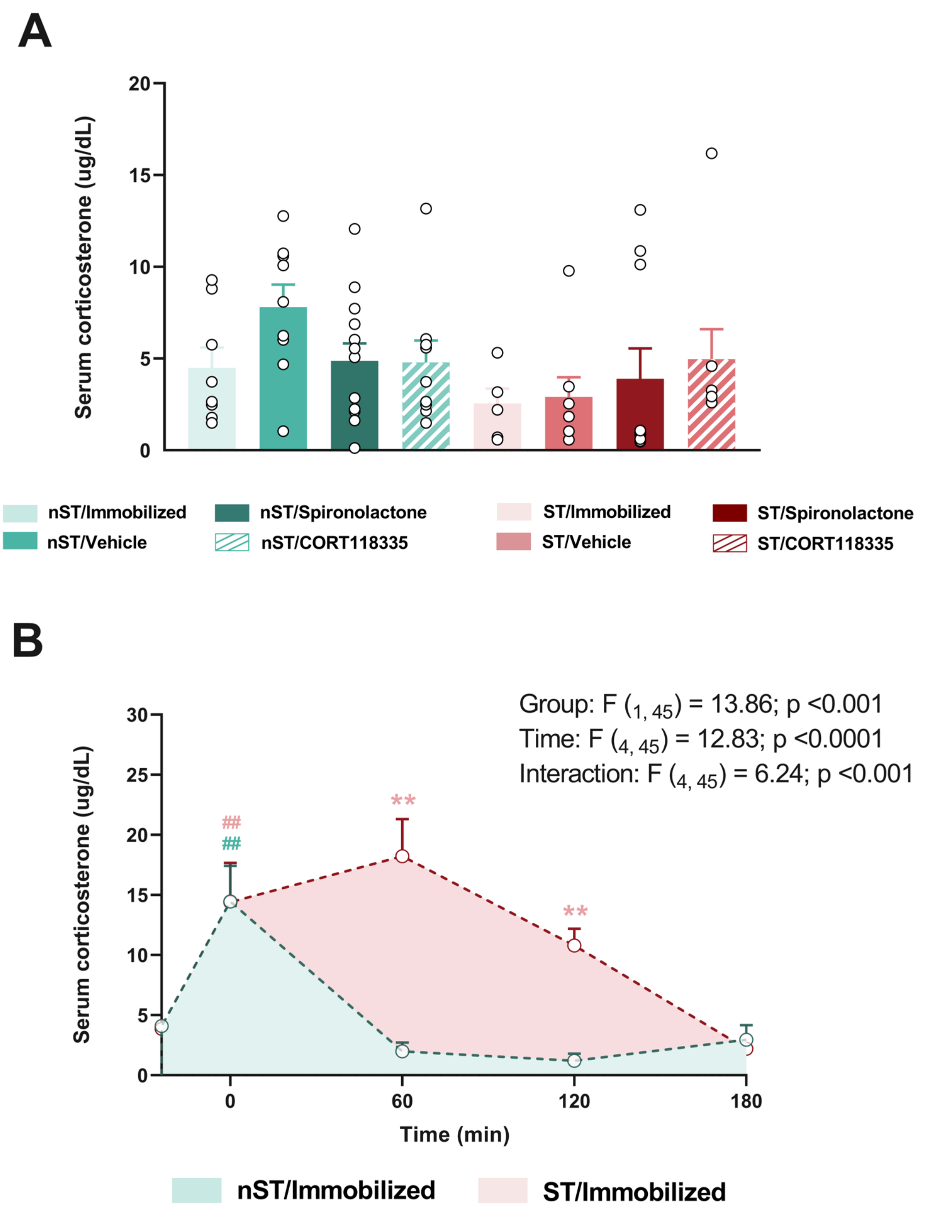

Corticosterone concentration in unstressed (nST) and stressed (ST) animals treated by spironolactone or CORT118335 indicates baseline similarity between groups (**A**). Corticosterone concentration curve in unstressed (nST) and stressed (ST) animals by restraint after 5 minutes of gentle immobilization (**B**). Results are represented as mean ± SEM (n = 7-13 animals per group in A; n = 5-8 animals per group in B). One-way ANOVA and two-way mixed-effects model ANOVA followed by Tukey's tests (A e B). Spironolactone and CORT118335 at dose 10 ng.μL^{-1}.
